# Oral Contraceptive Use and Breast Cancer Risk According to Molecular Subtypes Status: A Meta-Analysis

**DOI:** 10.1093/eurpub/ckac131.208

**Published:** 2022-10-25

**Authors:** S van Weers, R Hrzic, RJJ Elands

**Affiliations:** 1 Department of International Health, Maastricht University, Maastricht, Netherlands; 2 Historical Demography and Family History, Radboud University, Nijmegen, Netherlands

## Abstract

**Background:**

Breast cancer is a heterogeneous disease with distinct molecular signatures of disease etiology, evidenced by the joint expression of molecular tumor markers. Differential effects of oral contraceptive use on breast cancer risk by molecular subtypes have been reported. This is the first meta-analysis to investigate associations between oral contraceptive use and subsequent breast cancer risk stratified by combined estrogen receptor (ER) and progesterone receptor (PR) status alongside the Luminal A and B subtypes, which additionally consider the human epidermal growth factor receptor 2 (HER2) status.

**Methods:**

A systematic review and meta-analysis of case-control and cohort studies was conducted in PubMed and Web of Science. The odds ratios (ORs) were summarized using a random-effects model.

**Results:**

Eleven studies met the inclusion criteria for qualitative and quantitative analysis. Random effects meta-analyses revealed significant risk increasing effects for ever-users of oral contraception on ER-PR- breast cancer compared to never-users (OR = 1.30, 95% CI; 1.07 to 1.56, p < 0.01). Ever-use of oral contraception was not associated with breast cancer risk when stratified by the ER+PR+ breast cancer subtype (OR = 1.00, 95% CI; 0.86 to 1.16, p = 0.99). Data on Luminal A and B subtypes was limited and not suggestive for associations with breast cancer risk in ever-users of OCs compared to never-users. Furthermore, a significant increased risk of ER-PR- breast cancer was observed for OC use duration of > 4 years compared to never-users (OR = 1.74, 95% CI: 1.15 to 2.63, p < 0.01).

**Conclusions:**

The current state of the evidence suggests that OC use longer than 4 years is associated with an increased breast cancer risk, pertaining to the estrogen and progesterone double negative breast cancer subtype. Large-scale prospective observational studies including more comprehensive molecular signature of breast cancer aetiology, including HER2 status, are needed.

**Key messages:**

• The use of oral contraception was associated with estrogen and progesterone receptor double negative breast cancer, but not hormone receptive positive cancer.

• This is the first meta-analysis to investigate oral contraceptive use and associations with breast cancer risk by combined estrogen and progesterone receptor status.

